# Long-Term Outdoor Reliability Assessment of a Wireless Unit for Air-Quality Monitoring Based on Nanostructured Films Integrated on Micromachined Platforms

**DOI:** 10.3390/s120608176

**Published:** 2012-06-13

**Authors:** Matteo Leccardi, Massimiliano Decarli, Leandro Lorenzelli, Paolo Milani, Petteri Mettala, Risto Orava, Emanuele Barborini

**Affiliations:** 1 Tethis spa, via Russoli 3, Milano 20143, Italy; E-Mail: matteo.leccardi@tethis-lab.com; 2 Fondazione Bruno Kessler (FBK), via Sommarive 18, Povo (Trento) 38123, Italy; E-Mails: mdecarli@fbk.eu (M.D.); lorenzel@fbk.eu (L.L.); 3 CIMAINA and Physics Department of University of Milan, via Celoria 16, Milano 20133, Italy; E-Mail: pmilani@mi.infn.it; 4 Sensor Center Ltd.—Anturikeskus Oy, Pakkalankuja 5, Vantaa 01510, Finland; E-Mails: petteri.mehtala@sence.fi (P.M.); risto.orava@sence.fi (R.O.)

**Keywords:** gas sensors, metal-oxides, nanomaterials, micromachining, air monitoring, wireless network

## Abstract

We have fabricated and tested in long-term field operating conditions a wireless unit for outdoor air quality monitoring. The unit is equipped with two multiparametric sensors, one miniaturized thermo-hygrometer, front-end analogical and digital electronics, and an IEEE 802.15.4 based module for wireless data transmission. Micromachined platforms were functionalized with nanoporous metal-oxides to obtain multiparametric sensors, hosting gas-sensitive, anemometric and temperature transducers. Nanoporous metal-oxide layer was directly deposited on gas sensing regions of micromachined platform batches by hard-mask patterned supersonic cluster beam deposition. An outdoor, roadside experiment was arranged in downtown Milan (Italy), where one wireless sensing unit was continuously operated side by side with standard gas chromatographic instrumentation for air quality measurements. By means of a router PC, data from sensing unit and other instrumentation were collected, merged, and sent to a remote data storage server, through an UMTS device. The whole-system robustness as well as sensor dataset characteristics were continuously characterized over a run-time period of 18 months.

## 1. Introduction

Environmental sensing based on a network of small and inexpensive wireless sensors (motes) scattered over a very large area and able to detect tiny amounts of chemicals in air, water and soil has gained vast popularity among the scientific community and even in the popular press as a first line of defense to prevent terrorist attacks or environmental disasters [1]. Wireless sensor networks are also of interest for issues related to production and storage in the agro-food industry or other industrial processes requiring atmosphere control, air quality mapping in metropolitan area and large infrastructures, microclimatic condition monitoring for improved preservation of cultural heritage sites [2].

In pervasive sensing the information is extracted from the sensor network as a whole, in the form of a space distribution of a certain variable (temperature gradients, distribution of chemicals, wind velocity vector field, *etc.*) which can in principle be mapped and correlated with topological information such as for example, building distributions, the structure of main traffic-jammed roads, the presence of parks and rivers, industrial or dumping sites, *etc.* This requires that each node of the sensing network be characterized by the coexistence on the same unit of sensing capabilities, dedicated electronics, power supply and a wireless communication system [3].

Miniaturization is a key factor assuring limited power consumption, easiness of field installation and low cost. During the last two decades, silicon micromachining techniques have been applied to the fabrication of chemical and physical sensor microdevices, through the development of platforms with different structures and geometries (see, for example [4–8]). Micromachining techniques allow the integration on the same platform of different transducers for sensing, up to the electronics for signal processing (see for example [9,10]).

One of the most popular micromachined platform for gas sensors is the so-called micro-hotplate [11–13]. It consists of a multi-layer suspended membrane, having micrometric thickness and surrounded by a silicon rim, micromachined from a silicon wafer. The multi-layer membrane is typically composed by metallizations and several insulating layers, alternating each other, with various shapes. Metallic electrodes on the surface of the platform allow the readout of the chemoresistive signal from the metal-oxide sensing layer deposited on the top. Integrated heating elements provide sensor operation temperatures up to 500 °C. Due to its small mass and surface, as well as reduced thermal contact at border, the micro-hotplate requires very low heating power compared to traditional sensor platforms. Moreover, small thermal inertia allows the micro-hotplate to undergo quick thermal cycling, providing the possibility to operate sensing measurements with elaborated temperature protocols [14,15].

Despite these interesting characteristics, the effective use of micro-hotplates for gas sensing outside research labs is still hampered by the lack of a reliable and cost affordable method for large-scale integration of porous metal-oxide sensing layers, which is at the same time compatible with the mechanical delicacy of micro-hotplates, as clearly reported in [16]. Among various deposition techniques, it has been recently demonstrated that nanostructured metal-oxide layers with nanoscale porosity can be easily and safely integrated onto micro-hotplates by supersonic cluster beam deposition (SCBD) [17,18]. This approach seems to offer a way to overcome the main limitation affecting the large-scale use of micro-hotplates in gas sensing field (and micromachined platforms requiring nano-functionalization, in general).

Regarding real applicative scenarios, it has to be noted that besides an extremely voluminous literature on the characterization of micromachined gas sensor performances in terms of sensitivity, selectivity, response time, *etc.*, reports about the long-term stability and robustness under outdoor operation conditions are very scarce. This is quite surprising since the characterization of outdoor performances of wireless sensor nodes is a fundamental requisite to assess the feasibility of a sensor network.

Here we will report on the fabrication of multiparametric sensors consisting in a micromachined platform, where the metal-oxide layer for gas sensing is produced by SCBD in form of a nanostructured film. This platform is integrated with a miniaturized off-the-shelf thermo-hygrometer, proper front-end, pre-elaboration, as well as wireless communication electronics, in a wireless sensing unit. We will also report on the setup and results after 18-months of running of the outdoor experiment involving a wireless sensing unit, side by side with standard instrumentation for air quality monitoring, whose aims were at first the testing of the overall robustness of the sensing unit, and secondarily the comparison between gas sensing signals, as generated in multiparametric sensors by complex chemical composition of unconditioned free air of downtown Milan, and data from standard instrumentation for air quality monitoring.

## 2. Fabrication and Testing of the Gas Sensors

### 2.1. Micromachined Platforms

**Figure 1.**
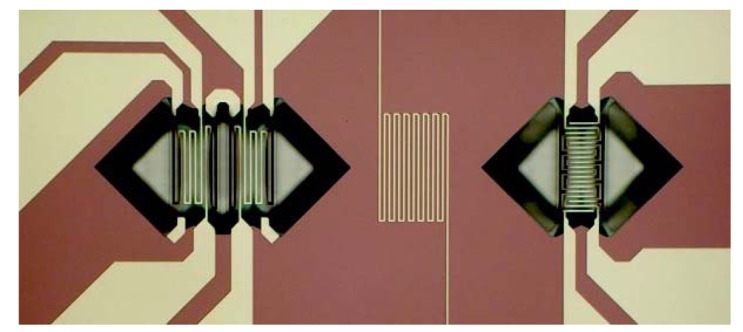
Image of the multiparametric sensing platform showing, from left to right, the anemometric transducer, the thermometer, and the gas sensor (the sensing layer is not present yet). Metal parts and dielectric parts appear with lighter and darker colors, respectively. Micromachining process adopted front-side etching approach, leading to suspended structures with “bridge” shapes. Black parts are the regions where silicon has been removed generating the bridges, while “lighter triangles” appearing at bridge sides are just optical effect: they also are etched regions. The overall size of the platform is about 3 × 5 mm^2^.

The sensor platforms of the present work have been developed with micromachining techniques in order to provide multiparametric sensors equipped with transducer for air temperature, air velocity, and gas detection, as shown in Figure 1. The transducer for air temperature is a Pt-wire thermometer deposited as a thin film with serpentine shape on the bulky central portion of the platform. Due to their high temperature operations, the anemometer and the gas sensor are both supported by micro-hotplates (more precisely, micro-hot-structures having “bridge shape” instead of “plate” shape). The anemometer flow sensor is composed by a heating element with two temperature sensors up- and downstream: the air flow causes anisotropic heat distribution that is measured by the mismatch of the two temperatures at heater sides. The gas sensor is realized with a microbridge having a heater and two interdigitated electrodes for the read-out of sensing layer resistance. Both the anemometer and the gas sensor microbridge structures were obtained by a micromachining route adopting front-side etching [18].

All metal structures are made of Cr/Pt (5 μm/250 μm) layer, which is known to be very stable for high temperature operation [19]. In the following, an indication of the resistance values of the platform elements is reported. Thermometer resistance at room temperature is about 2 kΩ, with 4 Ω/°C variation. Anemometer Pt heater and side Pt thermometers have a room temperature resistances of about 300 Ω, increasing to about 450 Ω at typical operation temperature of 250 °C, within static atmosphere. Power on heater is about 20 mW. Starting from a room temperature resistance of about 150 Ω, at operation temperature of 300–350 °C the gas sensor heater increases its resistance to 200–250 Ω, depending on heater material. Power on heater is about 40 mW. These features highlight the main differences with respect to commercial devices based on alumina platforms, whose purpose is limited to chemical sensing (they do not include other transducers), and power consumption for heating is as high as several hundreds of mW.

### 2.2. Nanoporous Sensing Layer Deposition

The deposition of the nanoporous metal-oxide layer over the region of the platform dedicated to gas sensing was carried out by SCBD, adopting a pulsed microplasma cluster source (PMCS). The method has been widely described elsewhere [20,21]. In summary, nanoparticles generated by means of an electrical discharge are directly transported toward deposition substrate by a carrier gas undergoing nozzle supersonic expansion. Once nanoparticles reach the deposition substrate, the growth of a nanoparticle-assembled layer takes place. The entire process is performed at room temperature and it is mechanically delicate for safe functionalization of micromachined parts, as other gas-phase deposition methods.

**Figure 2.**
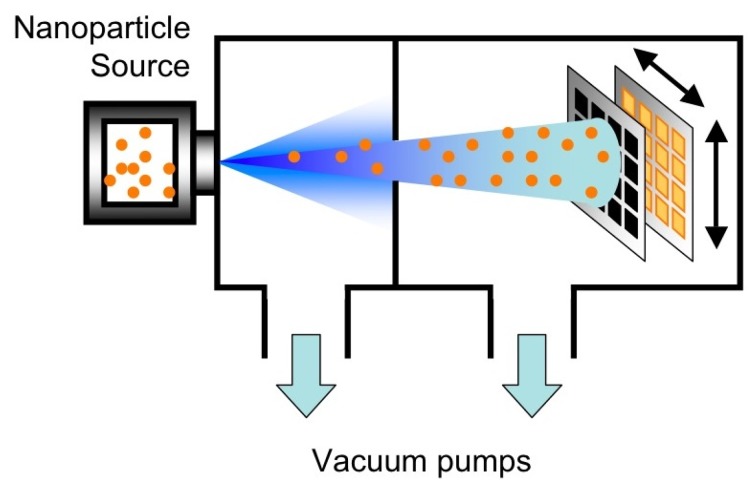
Schematic image of the apparatus for supersonic cluster beam deposition. It consists of a vacuum system, where nanoparticles are first generated by suitable sources, then immediately launched to deposition chamber by an expanding gas jet. In the present work, nanoparticles are generated by a PMCS. As schematically shown, beam collimation allows deposition patterning by non-contact hard-mask; deposition of many devices in batch is achievable by substrate rastering (as indicated by arrows).

**Figure 3.**
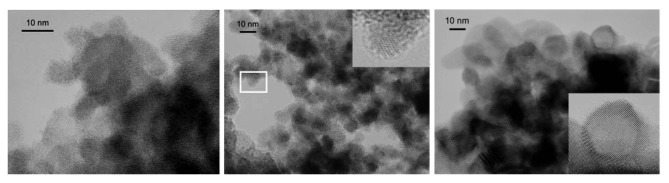
Structure of the metal-oxide sensing layer at the nanoscales, as resulting by Transmission Electron Microscopy (TEM) characterization. As-deposited material (**left**) shows the nanoporous structure generated by soft-assembly of nanoparticles with size below 10 nm. No lattice fringes are visible inside nanoparticles, indicating amorphous structure. After 200 °C annealing (**middle**), material structure evolves towards crystalline order, while particle size and overall porous structure are preserved. After 400 °C annealing (**right**), the crystalline fraction becomes predominant; the porous structure still appears substantially unchanged. Reprinted from [17].

Figure 2 schematically shows the deposition method. SCBD is characterized by a kinetic energy of nanoparticles as low as few tenths of eV per atom that prevents nanoparticles destruction at the impact with the substrate. Nanoparticle soft assembling generates highly porous, high specific surface area films, very well suited for applications where the interaction with the atmosphere has to be favored, such as in gas sensing. Figure 3 shows an example of a nanoparticle-assembled layer by SCBD. In addition, a wide library of nanostructured metal-oxides, including SnO_2_, TiO_2_, WO_3_, Fe_2_O_3_, MoO_3_, ZrO_2_, HfO_2_, NbO_x_, ZnO, PdO_x_, can be routinely produced by SCBD systems equipped with PMCS [22]. Although in the present experiment the materials used for the preparation of the sensors were limited to tungsten oxide and iron oxide, the nanomaterial library available discloses the perspective of producing complex sensing arrays.

Exploiting non-contact, hard-mask patterning we deposited the nanoporous sensing layer on the platform region dedicated to gas sensing only, avoiding any contamination of other regions. Batches of several tens of devices were deposited at the same time by sample holder rastering, which consists in up-down, left-right scans of the device batch in front of the nanoparticle beam. Delicacy of gas-phase deposition providing mechanical safety with respect to micromachined parts, one-step method for patterning the deposition over desired areas, absence of solvent/paste contamination, batch deposition of many devices per run, are in summary the main features of SCBD method, making its use for functionalization of micromachined parts particularly interesting.

After deposition, devices underwent thermal treatment in air at 450 °C in order to let oxides reach the proper stoichiometry and to induce any structure modification that may occur up to those temperatures. This step should “freeze” the sensing material structure and avoid any drift related to material modification during sensor operation, whose temperature is typically 300–350 °C.

**Figure 4.**
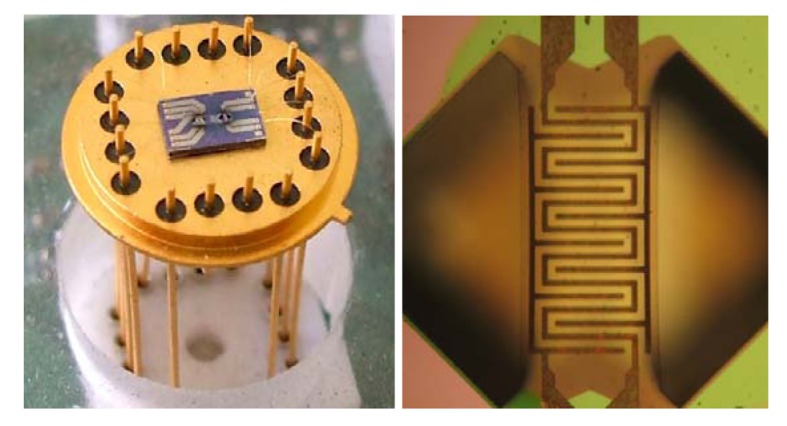
Image of the device, once micromachined platform has been bonded to TO package (**left**). A detail of gas sensing region of the platform is also shown (**right**): the greenish area is the active nanoporous layer (in this case, iron-oxide).

Regarding nanoporosity, it has been demonstrated that no substantial change occurs during this treatment [17]. Device preparation concludes after proper bonding to 16-pins TO package (Schott). Figure 4 shows the bonded device; in the inset, a detail of the gas sensing region of the platform is shown. Proper caps were chosen according to the characterizations to be carried out on the device: tube-shaped cap was adopted for lab-scale testing of anemometer performances (and anemometer in combination with gas sensor); a suitable Teflon cap, equipped with proper holes to favor gas diffusion toward the sensing platform, was adopted for outdoor experiments.

### 2.3. Front-End Electronics

Beside temperature transducers, whose reading does not pose any particular difficulty on front-end electronics due to their non-unusual resistance values, gas sensors require performing circuitry and careful structuring of the board. Huge resistances of the nanoporous metal-oxide sensing layers, with values up to several tens of GΩ, have to be handled. Moreover, in presence of reactive gases resistance values may undergo changes as large as two orders of magnitude in a few seconds. Hence, front-end electronic of gas sensor is required to manage high resistance values, changing with large dynamics. To this purpose, we developed a front-end based on ultra-low bias current operational amplifiers (Texas Instruments OPA129), providing linear or logarithmic reading of metal-oxide sensing layer resistance. The TO pins carrying gas sensing signal were isolated from the board and wired directly to Op. Amp. IN pin, to avoid as much as possible that any leakage current supported by board surfaces would affect the measure.

### 2.4. Lab-Scale Device Testing

**Figure 5.**
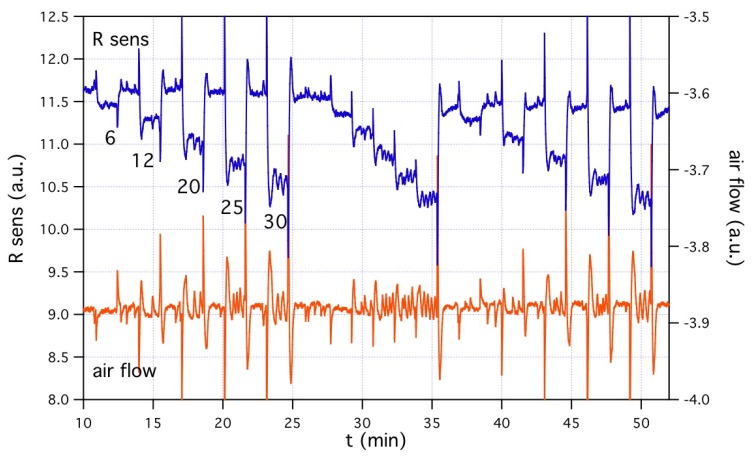

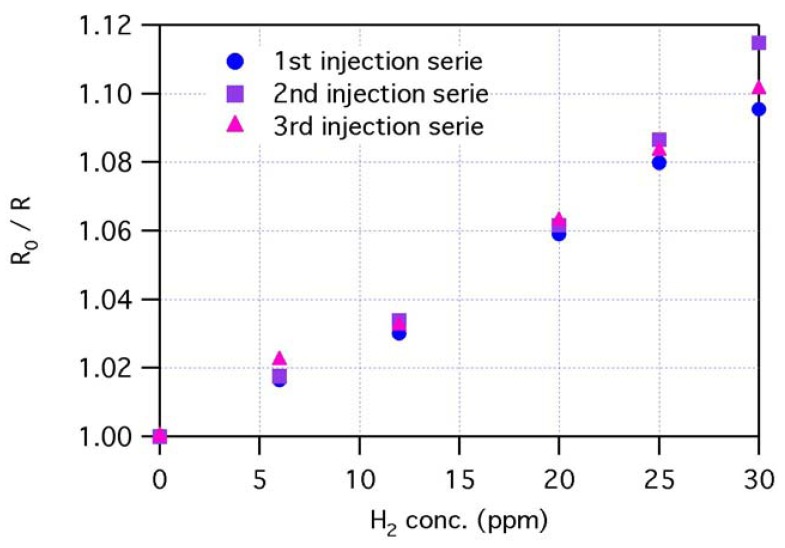
Top graph: example of device characterization, where gas sensing is performed contemporarily to anemometric air-flow measurements. Gas sensing graph is the blue one, referring to left axis; air-flow graph is the orange one, referring to right axis. The testing gas was hydrogen, at the labeled concentrations; both the chemical sensor and the anemometer were operated at 200 °C. Air-flow measurements require in-line insertion of device along gas pipeline. This condition gives the device the possibility to see, on both the gas sensor and the anemometer, real fluctuations -of chemical composition as well as of flow- due to the action of mass flow controllers of testing facility, as clearly appear in both graphs. Bottom graph: sensor response based on data of top graph (see text).

Before the outdoor experiments, devices underwent lab-scale characterization within a testing facility for gas sensors. The facility structure is basically constituted by a chamber, where atmosphere composition is controlled by means of two- or more- automatically operated mass flow controllers (MFCs), one dedicated to pure air (baseline atmosphere), others to gases to be added to baseline atmosphere and used for device response testing. Once concluded the characterization of gas sensing only, anemometric and gas sensing transducers were operated and tested contemporarily. The top graph in Figure 5 shows an example of these contemporary anemometric-gas measurements, using hydrogen. Gas injection was operated in order to determine the labeled concentrations, from 6 to 30 ppm. Although kept constant during each single gas sensing experiment, total flow in the range 260–360 sccm was used. Gas sensor and anemometer were operated both at about 200 °C.

To ensure proper meaning to anemometric air-flow measurements, a suitable tube-shaped cap was adopted and connected to gas line from MFCs. It is worth to highlight that, in this case, the absence of the test chamber, that acts as a gas buffer volume, allowed devices to unveil the dynamics of test atmosphere changes, related to real MFC operations, and gave us the opportunity to evaluate response and recovery dynamics of the gas sensor. Since no differences exist among gas dynamics as seen by the anemometer and by the gas sensor, even in the cases of sharpest structures, we conclude that gas sensor is not affected by some intrinsic delay mechanism slowing down its behavior, and may be used for the real-time monitoring of atmosphere, at least down to dynamics as fast as few tens of seconds.

The bottom graph of Figure 5 shows the response of the sensor to hydrogen, as calculated on the data of top graph: R_0_ is the baseline resistance value, *i.e.*, in absence of hydrogen, while R is the resistance value at a given hydrogen concentration. Data from “single step” first injection, “increasing steps” second injection, and “single step” third injection are included. In the case of “single step” injections, R_0_ values are the ones just before the injection; in the case of “increasing steps” injection, R_0_ is the value just before the first step. In all cases, the values used for calculations are the mean values over the time interval of each nominal concentration, excluded the first peak due to concentration change. Response dataset shows very good repeatability of chemical sensor at lab-scale testing, in the case of hydrogen at concentrations of 6–30 ppm.

## 3. Outdoor Experiment

### 3.1. Wireless Sensing Unit Structure

**Figure 6.**
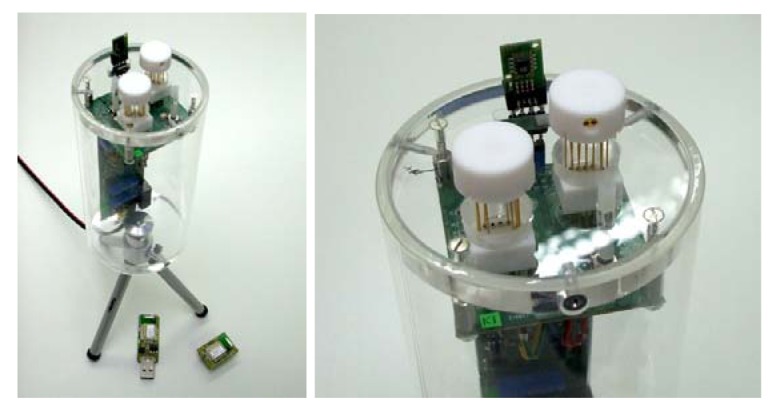
Images of the sensing unit. Blue socket hosts the wireless communication module, appearing at the base of the unit together with its USB wireless router, to be connected to the router PC.

As shown in Figure 6, the sensing unit for outdoor experiment has a cylindrical can-size structure, with a sensing head on top that hosts two of the mutiparametric devices described above and one miniaturized thermo-hygrometer (Sensirion SHT71). Multiparametric devices were horizontally positioned on sensing head plan with a 90° angle between them, in order to provide the chance to use anemometers to characterize in-plan air velocity components. Moreover, the use of two devices with different metal-oxides as sensing layers may enrich the information concerning gas sensing.

The sensing head board is orthogonally connected to the main board, that hosts analogical front-end electronics, CPU, and wireless data transmission components. CPU and dedicated firmware manage on one side the sensing devices, while on the other side communicate with a PC. The managing of sensing devices includes: the control of the heaters of anemometers and gas sensors (through heating power monitoring); the reading of the resistances of all thermometers; the reading of the resistances of metal-oxide sensing layers; and the data acquisition from thermo-hygrometer. The communication to PC may be performed by USB port, for lab-scale check and testing, or by wireless transmission, during outdoor experiment. Among many wireless communication modes, we chose a 6LoWPAN system (Sensinode Nanomodule N100, based on Radiocrafts RC2300 component), whose auto-configuring structure allowing data bouncing among nodes is particularly suited for sensor network outdoor operations.

### 3.2. Experimental Setup

The purpose of the outdoor experiment was first to check the robustness of the whole system, from the point of view of the reliability and stability of sensing signals, of micromachined platforms in itself, of electronics, of data logging process, *etc.*, during a long-term uninterrupted run, at open air. Or, in other words, to highlight the points of weakness. The second purpose was to collect data from the wireless sensing unit and, at the same time, from standard gas chromatographic (GC) instruments currently adopted for air quality characterization, to study dataset characteristics and search for correlations among them. The outdoor experiment was not intended to target a specific gas, rather it was intended to exploit the unspecificity of chemoresistive metal-oxide sensors to sense a multi-component, complex atmospheres such as that of metropolitan air.

**Figure 7.**
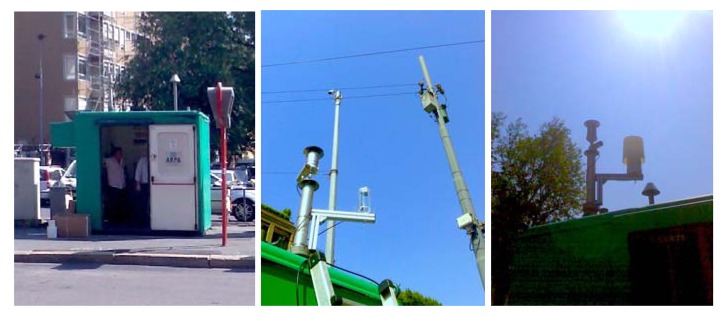
Outdoor experiment in downtown Milan. A station for air quality monitoring hosting standard GC instrumentation was selected as experimental site. Sensing unit was installed on the roof of the station, while the router PC collecting either the data from sensing unit, and the data from GC instruments, was installed in the inner area.

In collaboration with ARPA Lombardia (the regional agency in charge of air quality measurements), the wireless sensing unit was installed on the roof of one of the ARPA stations located downtown Milan, as shown in Figure 7. A position close to the access port of the tube collecting the air for GC instruments was chosen, in order to ensure the interaction with exactly the same atmosphere for both. A simple “mushroom-head”-like plastic cap was adopted to protect sensing unit from the rain. Power supply was wired, communication was wireless toward a PC equipped with a 6LoWPAN data router, which was located few meters down, into the station box. During the experiment gas sensors were continuously operated at 300 °C.

Through an A/D converter (National Instruments USB-6009 DAQ), PC also collected data from standard GC instrumentation, namely the concentration in air of: NO, NO_2_, CO, benzene, toluene, and xylene. Data from the sensing unit included: gas sensing and temperature from the two micromachined devices, temperature and relative humidity from the thermo-hygrometer. Data request frequency was set at one per minute. This parameter defines a limit with respect to dynamics of the phenomena to be characterized by the sensing system: in the case of outdoor atmosphere monitoring, one sample per minute may be sufficient (although a closer sampling could be easily managed, if needed). Data packages were sent by email to a data storage server once per hour, exploiting a commercial UMTS device for remote connection to Internet. The experimental setup also included a small free-standing uninterruptible power supply (UPS), to avoid PC and sensing unit failures due to power line troubles.

### 3.3. Results after 18th Month Running

According to the purposes of the present experiment, results after 18 months of operations are here divided between those regarding system robustness, and those regarding sensing unit data. A huge amount of meaningful data was collected, and various approaches and tools can be exploited for data analysis. We will limit here to show an overview of data general characteristics, as well as some relevant examples of specific features. At the end of the section, we will briefly report on one of the possible approaches to data analysis by means of Neural Network algorithms.

Concerning system robustness, the results may be summarized as follows: although various power line failures occurred at the ARPA station site during the experimental run (particularly during intense storms), no faults of the sensing unit and data router PC occurred thanks to the UPS. Data transmission out from the router PC suffered several faults due to disconnection of the UMTS device; this however did not result in data loss, due to the real-time data storage on the router PC. Wireless connection between sensing unit -on the roof- and router PC-into station box-experienced temporary faults during intense storms, causing data loss. This would suggest that a partial data log into the sensing unit itself could be necessary. No troubles were registered on the electronics-firmware side, although no specific actions to protect the unit from humidity, temperature, dust, as well as insects, nor particular shielding or grounding solutions were adopted.

**Figure 8.**
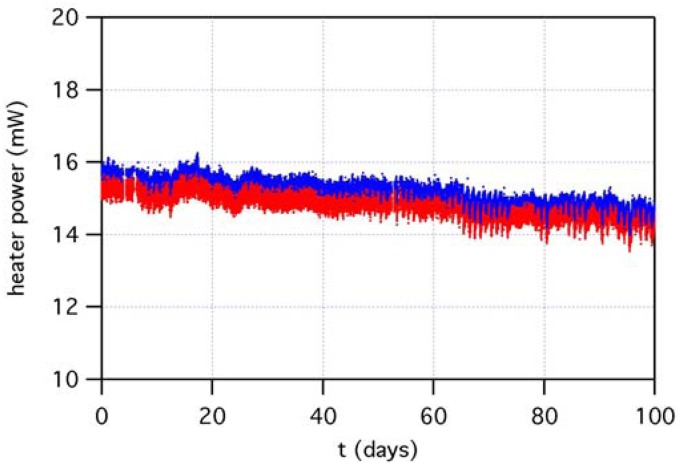
Heating power of both gas sensors, along the period between December 2010 and March 2011. The continuous -although slow- drift toward lower power unveils that some change in heater is taking place.

Robustness of sensor micromachined platform has been characterized through the monitoring of power on gas sensor heaters. In fact, the control of sensor operation temperature is performed by measuring the heater resistance, which depends on temperature, and by modulating the power accordingly. Any drift of the heather characteristics due to long-term, high-temperature operations would hence appear as a drift on power. Figure 8 shows heater power of both gas sensors, in the period between December 2010 and March 2011, as measured by the control system managing device temperature. Due to continuous operation at about 300 °C, the electrical characteristics along time of micromachined heater may be a meaningful indicator of the robustness of the platform, and of the underlying manufacturing technology. A continuous drift toward lower power is observed, indicating that a modification of the heater is taking place. In particular, the decreasing of the power may be explained with the increase of the resistance of the heater, at a given temperature: the control system associates higher resistance to higher temperature, hence decreases power. Although this behavior should result in some kind of drift also on gas sensing, no particular evidence of that appears from the gas sensing dataset (see below).

Regarding the characteristics of the various signals from multiparametric devices, we first observed that data from on-board thermometers dedicated to air temperature monitoring fit very well with temperature from Sensirion thermo-hygrometer (not shown).

**Figure 9.**
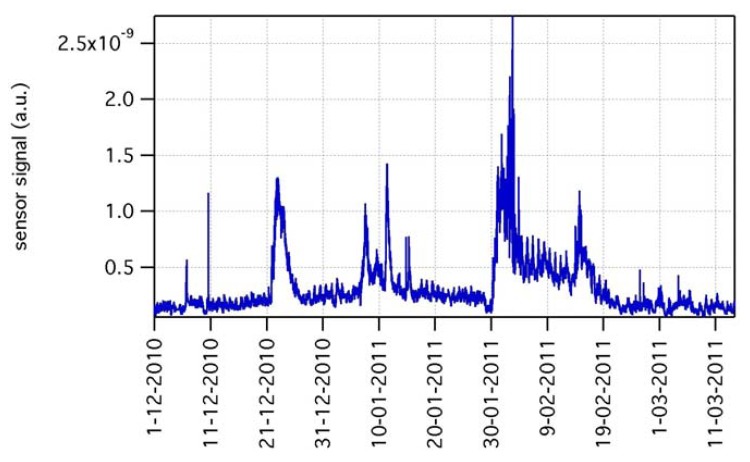
Gas sensing signal during outdoor experiment, in the period December 2010–March 2011.

**Figure 10.**
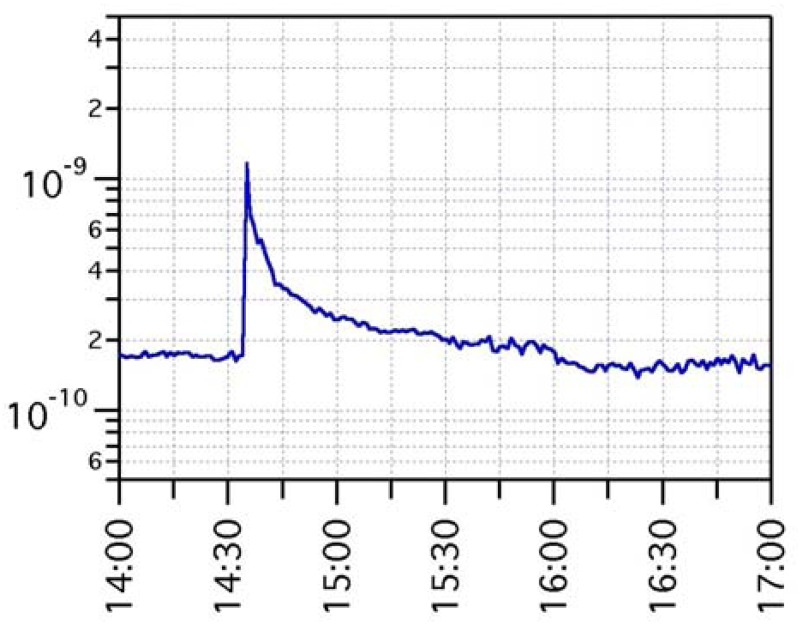
Zoom-in of 11-12-2010 event, appearing in Figure 9 as just a vertical line: as shown here, this is a real sensing event, with sharp onset and about 1 h recovery time. Vertical log scale emphasizes an almost tenfold change in sensing layer resistance value.

**Figure 11.**
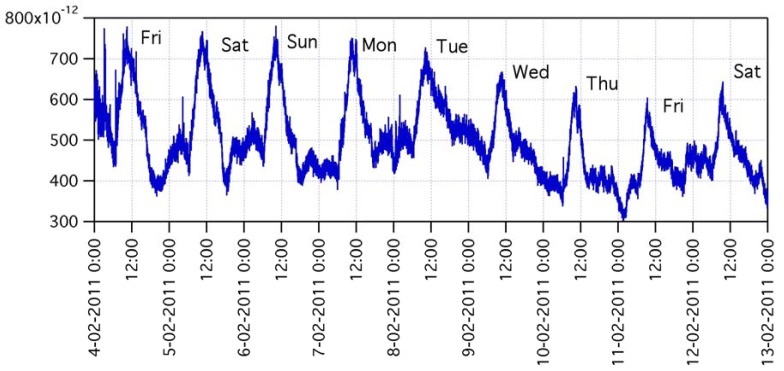
Zoom-in of the period between 04-02-2011 to 13-02-2011, showing day-night signal modulation.

The gas sensing dataset spanning over such a long period is very rich in features and dynamics. Events such as seasonal change (and related modulation of buildings heating systems), working days-weekend rotation (and related modulation of automotive emissions), Christmas and New Year's Day holidays (and related changes of downtown life), *etc.*, may all contribute to signal features. The same holds for single random events, such as big truck transit along the road or cars maneuvers in a car parking close to ARPA station. Provided these heterogeneous scenarios, non-selectivity of microsensors may play a key role in the detection of any change in the chemical composition of the atmosphere, no matter the specific gas that caused it. Figure 9 shows the signal of one of the gas sensors over the period December 2010–March 2011. According to accepted approach in the definition of the response of metal-oxide sensors to reducing compounds as R_0_/R, where R is the sensing layer resistance and R_0_ is a constant, the sensor signal is plotted as 1/R. The graph highlights the superposition of various structures: a rather regular and stable baseline, a day-night modulation, various long-lasting sensing events with many-days tails, and various short-lasting events with few-hours tails. Signal variations during sensing events, either long or short lasting ones, may be as high as more than one order of magnitude of baseline level. Figures 10 and 11 are zoomed-in portions of Figure 9, showing examples of one short-lasting event, and of a one-week-long day-night modulation, respectively.

**Figure 12.**
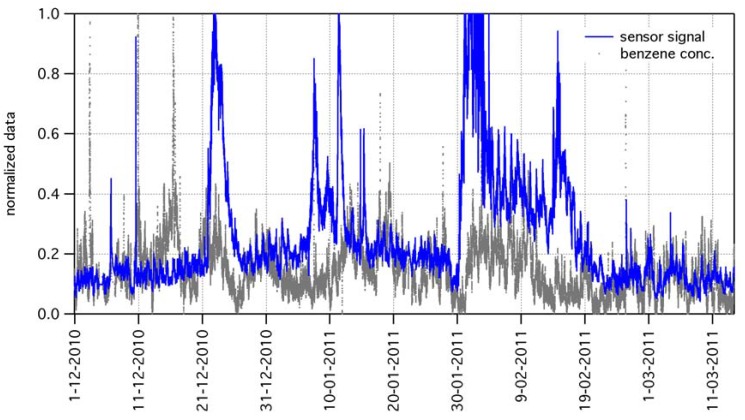
The graph shows, beside gas sensor signal (same data of Figure 9), the benzene concentration, as measured by GC instrument. In several parts of the graph, a rather good correlation between data clearly appears. However, various uncorrelated events can be also seen. This makes the deep comprehension of the origin of outdoor data features a definitely non-trivial task to be addressed.

Although the dataset includes measurements from GC instruments (concentrations of NO, NO_2_, CO, benzene, toluene, and xylene), as well as measurements of temperature and humidity, the correlation of gas sensor signal features to those variables is anything but a straightforward task. For example, Figure 12 shows the data of benzene concentration beside the gas sensor signal (same data of Figure 9, after suitable normalization).

Although in several parts of the graph a rather good correlation between GC data and gas sensor data clearly appears, various uncorrelated events also exist. This general indication in the case of benzene holds for all other compounds undergoing GC measurements. Therefore, simple comparisons fail to unambiguously associate gas sensor signal to signals from other instrumentations, suggesting that complex tool for data analysis should be adopted. Results of data analysis will be the subject of a forthcoming paper, however we anticipate here a brief description of the followed approach and the preliminary results.

Due to the fact that reference variables themselves (GC data, air temperature and humidity) at each point of time are not enough to accurately predict the output of the gas sensors at that point, we started by investigating if the short or long scale history of the sensors may play a role in affecting gas sensor output. Among various approaches in the use of advanced algorithms for data analysis taking into account history, we focused on Neural Networks (NN) [23]. A feedforward NN with backpropagation training algorithm using the gradient descent method was used. The training target was the gas sensor output signal, while the training inputs were the reference measurements (absolute humidity, temperature, NO, NO_2_, CO, benzene, toluene, and xylene). The architecture of the NN was arranged with an input layer with eight neurons, two hidden layers each with 20 neurons and output layer. The hidden layers and the output layer use logistic sigmoid transfer functions and linear transfer function, respectively.

The processed data consisted in over 300,000 measurement points of reference and gas sensor data; among those, 50,000 points were used for the training at a time. The training sample was divided randomly so that 60% was assigned to the training set, 20% to the validation set, and 20% to the test. The validation set was used to check how well the NN generalizes outside the training set, while the test set was used to show the performance of neural network.

Although the data analysis task is still running, we anticipate here that the performances of NN in predicting gas sensor data appear to be different for the two devices of the sensing unit, that use different metal-oxides as active sensing layer: signal of gas sensor using iron oxide is more predictable than the signal of the gas sensor using tungsten oxide. Moreover, even in the best case, some deviations between NN outcomes and sensor data can still be observed, suggesting that some variables not included in the group of reference data, play a role in gas sensor output. We do not show additional details here since the subject of data analysis will be deeply addressed in a forthcoming paper.

It is worth highlighting here that, on the one hand, GC instruments give precise measurements of the concentration of one specific gas, while, on the other hand, metal-oxide gas sensors may detect several compounds, due to their non-selectivity. If, at first glance, non-selectivity seems to be a limitation (and in many applicative scenarios it is), for air monitoring, where a plethora of different chemical compounds may play a role, it could turn out to be a resource: any event or any change respect to a “baseline situation” could be detected.

## 4. Conclusions

We have fabricated multiparametric sensors for air monitoring, exploiting nanoporous metal-oxide layers for the chemical sensing function. Nanoporous layers have been integrated on micromachined platforms through supersonic cluster beam deposition. Two of these devices and one miniaturized thermo-hygrometer were integrated into sensing units for air monitoring, equipped with suitable data-handling electronics and the module for wireless data transmission.

Long-term outdoor experiments gave information on system robustness as well as on the features of sensing data. Micromachined heaters, which were constantly operated at 300 °C, suffered a minor drift of their characteristics, however gas sensing data does not seem to be affected by such a drift. In addition, this also indicates that nanoporous metal-oxide sensing layers have good stability with respect to long-time operation at high temperature. Heater drift could be in principle reduced by operating the sensors in temperature-modulation mode.

After 18 months of outdoor operation, non-selectivity of metal-oxide gas sensors provided a very rich as well as complex dataset, where many sensing structures are overlapped. Comparison with GC measurements, contemporarily sampling the same atmosphere for the detection of NO, NO_2_, CO, benzene, toluene, xylene, indicates that metal-oxide sensors can detect events that escape standard instrumentation commonly adopted for air quality measurement. Data analysis by means of Neural Networks or other advanced algorithms may lead to a deeper comprehension of the origin of data features, and to a better evaluation of system potentialities in the context of air monitoring. In addition, the possibility to use several gas sensors, each one having a different metal-oxide sensitive layer, may offer a rather easy way to increase further the richness of the collected dataset, in view of advanced data processing.

From a general point of view, we have shown that the main elements composing a miniaturized sensing system for air monitoring (micromachined platform, nanoporous sensing layer, integrated electronics, wireless communication units, and proper algorithms for network and data managing) have reached a satisfactory development stage, as required for in-field applications. However it seems that a gap still exists between the potentiality of micromachined gas-sensors, as claimed at the laboratory level, and the demonstration of their effective use, in the form of pervasive sensing networks, in real-world applications. To our opinion, only long-term validation activities, where sensor networks are intensively tested in well-defined real scenarios, may definitely bridge this gap.
